# Complement C1q (C1qA, C1qB, and C1qC) May Be a Potential Prognostic Factor and an Index of Tumor Microenvironment Remodeling in Osteosarcoma

**DOI:** 10.3389/fonc.2021.642144

**Published:** 2021-05-17

**Authors:** Long-hao Chen, Jin-Fu Liu, Yan- Lu, Xin-yu He, Chi- Zhang, Hong-hai Zhou

**Affiliations:** ^1^ Faculty of Orthopedics and Traumatology, Guangxi University of Chinese Medicine, Nanning, China; ^2^ Graduate School, Guangxi University of Chinese Medicine, Nanning, China

**Keywords:** C1q (A/B/C), tumor microenvironment, osteosarcoma, tumor-infiltrating immune cells, CIBERSORT, ESTIMATE

## Abstract

The tumor microenvironment (TME) has important effects on the tumorigenesis and development of osteosarcoma (OS). However, the dynamic mechanism regulating TME immune and matrix components remains unclear. In this study, we collected quantitative data on the gene expression of 88 OS samples from The Cancer Genome Atlas (TCGA) database and downloaded relevant clinical cases of OS from the TARGET database. The proportions of tumor-infiltrating immune cells (TICs) and the numbers of immune and matrix components were determined by CIBERSORT and ESTIMATE calculation methods. Protein-protein interaction (PPI) network construction and Cox regression analysis were conducted to analyze differentially expressed genes (DEGs). The complement components C1qA, C1qB and C1qC were then determined to be predictive factors through univariate Cox analysis and PPI cross analysis. Further analysis found that the levels of C1qA, C1qB and C1qC expression were positively linked to OS patient survival time and negatively correlated with the clinicopathological feature percent necrosis at definitive surgery. The results of gene set enrichment analysis (GSEA) demonstrated that genes related to immune functions were significantly enriched in the high C1qA, C1qB and C1qC expression groups. Proportion analysis of TICs by CIBERSORT showed that the levels of C1qA, C1qB and C1qC expression were positively related to M1 and M2 macrophages and CD8+ cells and negatively correlated with M0 macrophages. These results further support the influence of the levels of C1qA, C1qB and C1qC expression on the immune activity of the TME. Therefore, C1qA, C1qB and C1qC may be potential indicators of remodeling in the OS TME, which is helpful to predict the prognosis of patients with OS and provide new ideas for immunotherapy for OS.

## Introduction

Osteosarcoma (OS), which accounts for 9% of cancer deaths in children and adolescents aged 10-24 years, is the most common primary malignant bone tumor in children and adolescents. It has strong local invasiveness and early metastasis, with signs of metastasis indicating a poor prognosis in advanced osteosarcoma patients (1, 2). At present, chemotherapy combined with surgical resection is the most important method of OS treatment. The disease-free survival rate of OS patients is close to 70% (3). The prognosis of patients with OS recurrence or metastasis is still extremely poor, although great success has been achieved with multimodal combination therapies. The five-year overall survival rate is approximately 20%, which is still insufficient to a large extent (4, 5). Therefore, identifying new diagnostic biomarkers and developing more effective molecular targets for cancer treatment are still warranted.

In recent years, increasing attention has been given to the effects of the tumor microenvironment (TME) on tumorigenesis and development (6). Immune cells and stromal cells are the main structural components in the TME. They provide all the metabolites and factors that control the proliferation, spread, and drug resistance of OS cells. Tumors are inextricably linked to their local microenvironment (7). Studies have shown that stromal cells are involved in tumor angiogenesis and extracellular matrix remodeling (8). The interactions between host stromal cells and tumor cells play key roles in the growth and progression of tumors. However, different tumors have different matrix components. The mechanism of crosstalk between tumors is still poorly understood (9). Furthermore, the impacts of immune cells in the TME on the tumor have also attracted much attention. Many studies have tried to define the interactions between the immune system and tumors, and it is possible to conduct therapeutic interventions based on these interactions. Studies have shown that TICs in the TME are an important indicator for OS treatment decision-making, and the infiltration of lymphocytes and macrophages is remarkably linked to the prognosis of OS patients (10, 11). Immune-related risk models for OS have been established in some studies for the prediction of OS patient prognosis. The results indicated that a poor prognosis and immunosuppression were common in patients with a high risk score (12). The development of tumor-immune correlation studies has led to continuous improvements in tumor immunotherapy. Therefore, accurate genetic analysis that correctly reveals the dynamic mechanism regulating TME immune and matrix components is a research focus and a challenge.

In this paper, ESTIMATE and CIBERSORT calculation methods were applied to calculate the proportions of TICs and TME immune/interstitial components in OS patient samples collected from the TCGA database and ultimately identified the complement family members C1qA, C1qB and C1qC as predictive biomarkers. C1q is not only a key subcomponent of the classical complement activation pathway but also the main link between innate immunity driven by the classical pathway and acquired immunity mediated by IgG or IgM. C1q is synthesized in the tumor microenvironment and acts as an extracellular matrix protein that can promote tumor growth and metastasis (13, 14). Some studies have shown that C1q expression is present in the microenvironments of diverse types of human tumors, including prostate cancer, ovarian cancer, mesothelioma, and melanoma. In these microenvironments, C1q may perform promotive or inhibitory roles in cancer progression, but the majority of these results indicate that C1q expression in the tumor microenvironment is linked to a poor patient prognosis (15). In this paper, starting from the differentially expressed genes (DEGs) identified by comparing TME immune components and matrix components in OS patients, it is revealed that the complement family members C1qA, C1qB and C1qC may be potential indicators of TME remodeling in OS patients.

## Data Collection and Processing

### Materials and Methods

The quantified HTSeq-FPKM gene expression data for 88 OS samples were downloaded from the TCGA on August 9, 2020. At the same time, the latest clinical data for OS patients, including age, sex, race, overall survival time, survival status and other clinicopathological information, were downloaded from TARGET (https://ocg.cancer.gov/programs/target).

To screen the mRNA matrix data with genetic characteristics, we used PERL software (v5.30.2) (https://www.perl.org/) to annotate the human genome based on the expression profiles of the OS tumor samples downloaded from the TCGA database. Matrix data for the gene expression values of the OS tumor samples were obtained.

### Establishment of the ImmuneScore, StromalScore, and ESTIMATEScore

Estimation of the levels of TME immune-stromal components in each sample was conducted using the ESTIMATE algorithm using R software (version 4.0.2) loaded with the estimate package (16). Three types of scores (ImmuneScore, StromalScore, and ESTIMATEScore) exhibited a positive association with 3 kinds of levels (immune, stromal, and the sum of both). The levels of the corresponding TME components were higher when the corresponding score was higher.

### Survival Analysis

The combination of the TME score with the survival and survival status of OS patients was conducted using the Limma package of R software. According to the median immune score and matrix score, OS samples were divided into high- and low-score groups. Survival analyses were carried out using R software with the packages survival and survminer. Survival curves were plotted by the Kaplan-Meier method, and statistical significance was determined with the log-rank test; *p* < 0.05 was considered statistically significant.

### Screening of DEGs Between the High and Low ImmuneScore or StromalScore Groups

The high and low ImmuneScore or StromalScore results for the 88 tumor samples were determined by comparing the results with the median score. Differential gene expression analyses were performed using R with the limma package, and DEGs were identified by comparing the high- and low-score samples. An FDR<0.05 and a fold change after log2 transformation >1 (high-score group/low-score group) were used to select the significant DEGs.

### GO and KEGG Enrichment Analyses

GO and KEGG enrichment analyses of 379 DEGs were conducted using R software with the packages clusterProfiler, enrichplot, and ggplot2. Terms were considered significantly enriched terms both the *p*- and *q*-values were <0.05.

### Heatmaps

R software with the package pheatmap was applied for the production of DEG heatmaps.

### Analysis of Score Differences in Clinicopathological Data

The clinicopathological data of patients with OS were statistically analyzed with R software, and comparisons of different clinical parameters were conducted with Wilcoxon or Kruskal-Wallis rank-sum tests to determine significance.

### Construction of a PPI Network

The STRING database and Cytoscape (version 3.7.1) were applied for the construction and reconstruction of a PPI network, respectively. The network was built using the nodes with an interactive confidence value >0.7.

### COX Regression Analysis

Univariate Cox regression analysis was conducted using R software with the survival package. The patients were divided into high and low expression groups based on the median gene expression value. The survival difference between the two groups was evaluated, and the Kaplan-Meier test was performed. The identified significant genes shown in the figures satisfied both univariate Cox *p* < 0.05 and Kaplan-Meier test *p* < 0.05.

### Gene Set Enrichment Analysis

GSEA was conducted using gsea-4.1.0 software on the basis of the Molecular Signatures Database-derived HALLMARK and C7 target sets (v6.2). GSEA was conducted using the whole transcriptome of all tumor samples, and only gene sets with an FDR q < 0.06 and a NOM *p* < 0.05 were considered significant.

### TIC Profile

Estimation of the TIC abundance profile among all tumor samples was carried out by the CIBERSORT computational method. The genes with low expression were removed, and a follow-up analysis was performed using only the tumor samples with *p* < 0.05.

## Results

### Analysis Process of This Study

We downloaded data for 88 RNA-SEQ transcriptomes from the TCGA database and corresponding clinical data from the TARGET database. The proportions of tumor-infiltrating immune cells (TICs) and the numbers of immune and matrix components in the TME of OS patients were determined by CIBERSORT and ESTIMATE calculation methods. A protein-protein interaction (PPI) network was constructed using DEGs identified by both ImmuneScore and StromalScore, and then univariate Cox regression analysis was carried out. Intersection analyses were then applied to analyze the PPI core nodes and most significant factors identified by the Cox regression analysis. Seven key genes, ITGAM, C1qB, CD163, C1qA, C1qC, C3AR1 and CD14, were identified. We continued to screen the relationships between gene expression and survival and ultimately identified C1qA, C1qB and C1qC as the focus of a follow-up series of analyses, including gene set enrichment analysis (GSEA) and correlation analysis of clinicopathological features and TICs.

### Scores Were Associated With OS Patient Survival

**Figure 1:**
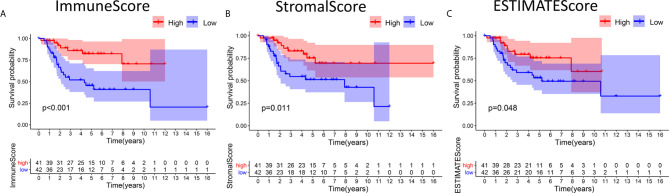
Associations between the scores and survival of OS patients. **(A)** Kaplan-Meier survival analysis of OS patients in the high and low ImmuneScore subgroups, log-rank test *p* < 0.001. **(B)** Survival analysis of patients in the high and low StromalScore groups, *p* =0.011. **(C)** Survival analysis of OS patients in the high and low ESTIMATEScore groups, *p* = 0.048.

Kaplan-Meier survival analyses based on ImmuneScore, StromalScore and ESTIMATEScore results were performed to clarify the relationships between immune and matrix levels and survival rates. The number of immune cells or stromal cells in the TME is considered to be greater when the ImmuneScore or StromalScore result is higher. The combined result for the ImmuneScore and StromalScore for the TME is the ESTIMATEScore, which is calculated as the sum of the two scores. **Figures 1A, B** shows that the contents of immune cells and stromal cells were markedly linked to OS patient overall survival. The correlation between the ESTIMATEScore and overall survival was also significant (**Figure 1C**). It is suggested that the composition of TME can predict the prognosis of patients with OS.

### Enriched DEGs Shared Between the ImmuneScore and StromalScore Were Mainly Immune-Related Genes

**Figure 2:**
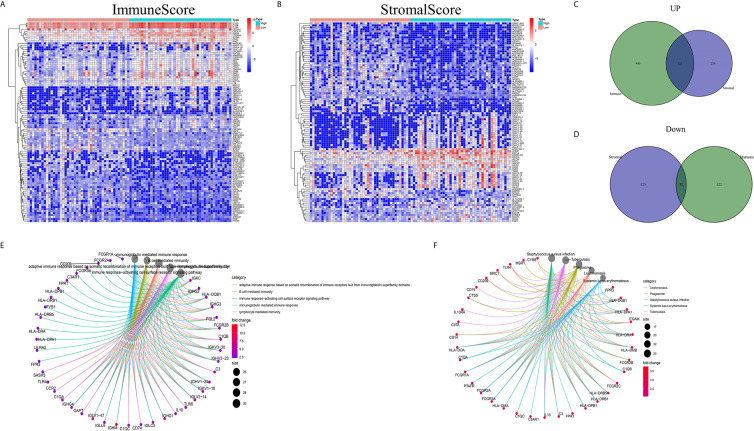
DEG heatmaps, Venn diagrams, and GO and KEGG enrichment analyses. **(A)** Heatmaps for DEGs identified by comparing the high-score and low-score ImmuneScore groups. The gene name is shown in the row, and the sample ID (column name) is not shown. The Wilcoxon rank-sum test was used to identify differentially expressed genes, and the threshold of significance was set at *q*=0.05 and a fold change after log2 conversion >1. **(B)** DEG heatmaps, similar to **(A)**, produced by analysis related to the StromalScore. **(C, D)** The Venn diagrams displaying the shared up- or downregulated DEGs identified by the analyses using the ImmuneScore and StromalScore, with a filtering threshold of significance of *q*<0.05 and a fold change after log2 conversion >1. **(E, F)** GO and KEGG enrichment analyses were carried out for the 155 DEGs, with p and *q* < 0.05.

Analyses comparing samples with high and low scores were conducted to determine the exact gene profile alterations in immune and stromal components of the TME. In total, 1163 DEGs (609 upregulated and 554 downregulated genes) were acquired from the ImmuneScore (high- and low-score samples) compared to the median (**Figures 2A, C, D**). Similarly, 934 DEGs (379 upregulated and 555 downregulated) were acquired from the StromalScore (**Figures 2B–D**). In addition, 123 high-score upregulated genes and 32 low-score downregulated genes in the ImmuneScore and StromalScore were shown to intersect by Venn diagram analysis. These DEGs (155 genes in total) may be determinants of the TME status. Enrichment analysis of gene ontology (GO) results demonstrated that the DEGs generally mapped to terms linked to immunity, such as immunity mediated by B cells or immunoglobulin (**Figure 2E**). Kyoto Encyclopedia of Genes and Genomes (KEGG) enrichment analysis also showed DEG enrichment in the immune system-related disease spectrum (**Figure 2F**). Therefore, mapping of the DEGs to activities linked to immunity suggested that the key feature of the TME in OS is immune factor involvement.

### Intersection Analysis of a PPI network and Univariate Cox Regression

**Figure 3:**
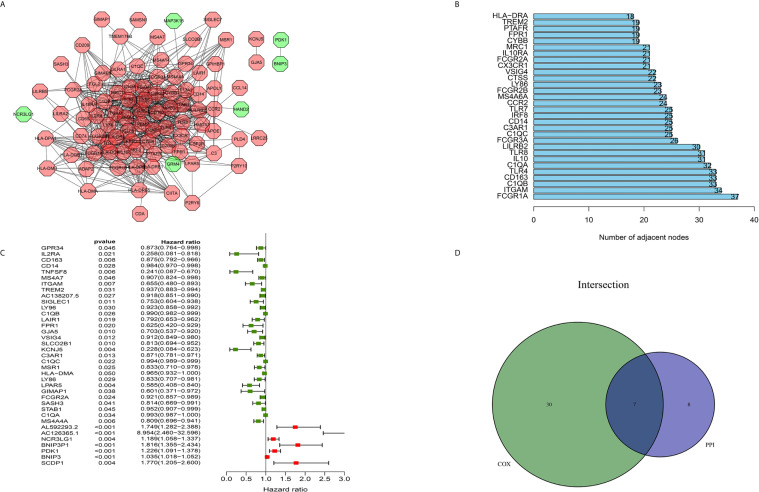
Construction of a PPI network, univariate analysis by Cox regression analysis and intersection of the results. **(A)** Construction of the PPI network conducted using nodes with an interactive confidence value > 0.7. **(B)** Sorting of the top 30 genes on the basis of the number of PPI nodes. **(C)** The 155 DEGs were analyzed using univariate Cox regression analysis and the Kaplan-Meier test, and significant influencing factors with P < 0.05 are listed. **(D)** Venn diagram constructed to identify the factors shared by the top 30 PPI nodes and the top 15 univariate Cox regression factors.

The construction of a PPI network with the STRING database was performed using Cytoscape software to further reveal the underlying mechanism of the OS TME. **Figure 3A** shows the interactions among the 155 genes, and the top 30 genes were sorted based on the number of nodes (**Figure 3B**). Determination of significant prognostic factors for OS patients was conducted using univariate Cox regression analysis of the survival of the 155 patients (**Figure 3C**). Analyses of the intersections between the PPI core nodes and the top 15 Cox regression factors were then performed, identifying 7 overlapping factors, namely, C1qA, C1qB, C1qC, C3AR1, CD14, CD163, and ITGAM (**Figure 3D**).

### Relationships Between DEG Expression and Survival Time in Patients With OS

**Figure 4:**
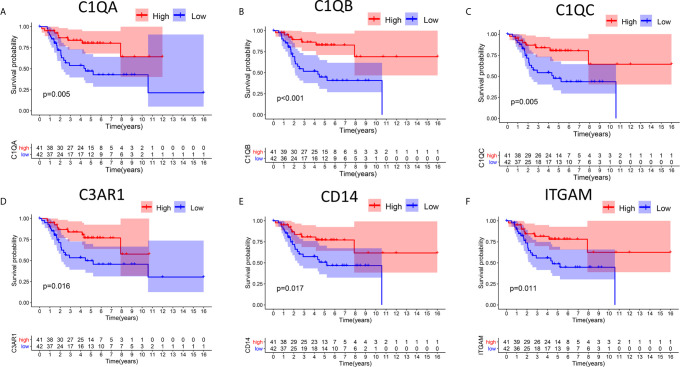
Relationships between DEG expression and survival time in patients with OS. **(A–F)** Differences in survival between patients with high and low expression of DEGs. The high and low expression levels of DEGs in patients were defined according to the median expression level. *p <* 0.05 (log-rank test) for all these genes.

In this study, high and low gene expression groups were established to divide all OS samples according to median gene expression levels. DEG survival analysis indicated that the survival rates of OS patients with high expression of C1qA, C1qB, C1qC, C3AR1, CD14 or ITGAM were higher than those of patients with corresponding low expression (**Figures 4A–F**). C1qA, C1qB and C1qC showed significant differences at the P<0.01 level. Therefore, we concluded that expression of C1qA, C1qB, C1qC, C3AR1, CD14 and ITGAM in the TME is positively correlated with the prognosis of OS patients, among which C1qA, C1qB and C1qC were more closely linked to the prognosis of OS patients. We identified C1qA, C1qB and C1qC as the main prognostic indicators in patients with OS.

### Relationships Between the Levels of C1qA, C1qB, or C1qC Expression and Clinicopathological Characteristics in OS Patients

**Figure 5:**
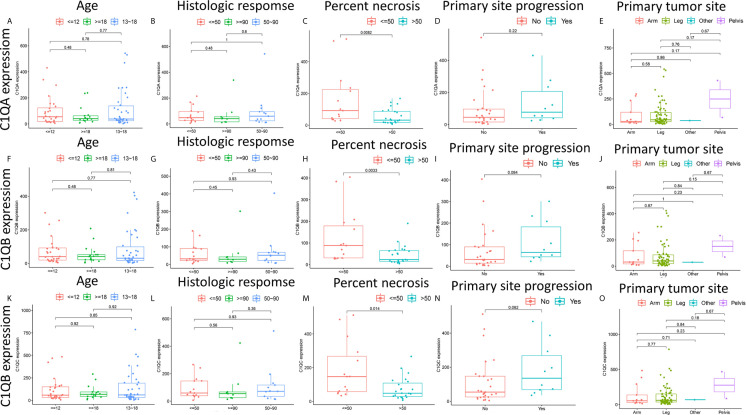
**(A–O)** Relationships between the levels of C1qA, C1qB, or C1qC expression and clinicopathological features in OS patients. The Wilcoxon rank-sum test or Kruskal-Wallis rank-sum test was applied for statistical analysis.

There are clear correlations between the levels of C1qA, C1qB, and C1qC expression and the survival time of OS patients. To further clarify the correlations between the expression levels of these factors and age, histologic response, percent necrosis at definitive surgery, primary site progression and primary tumor site in patients with OS, the relationships between the levels of C1qA, C1qB, or C1qC expression and these five clinical features of OS patients were analyzed. The results indicated that the levels of C1qA, C1qB and C1qC expression were significantly correlated with percent necrosis at definitive surgery in OS patients (*P* < 0.05) (**Figures 5C, H, M**), while age, histologic response, primary site progression and primary tumor site were not significantly correlated with C1qA, C1qB or C1qC gene expression (*P* > 0.05) (**Figures 5A, B, D–G, I–L, N, O**). We found that the C1qA, C1qB and C1qC gene expression levels in OS patients with a percent necrosis at definitive surgery less than 50% were higher than those in OS patients with a percent necrosis at definitive surgery higher than 50%.

### C1qA, C1qB, and C1qC May Be Potential Indicators of TME Remodeling

**Figure 6:**
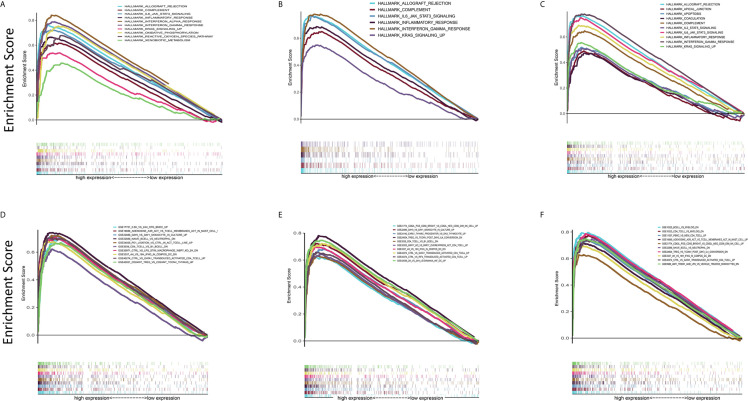
GSEA of samples with high and low C1qA, C1qB and C1qC expression. **(A–C)** C1qA, C1qB and C1qC are associated with the gene set enriched in HALLMARK. Each line represents a specific set of genes, with the upregulated genes located at the origin near the coordinates on the left and the downregulated genes located on the right side of the x-axis. FDR *q* < 0.05 and NOM *p* < 0.05 were considered significant. Only a few leading gene sets are displayed in the figure panels. **(D–F)** Expression of genes in the C7 gene set (immunological gene set) in samples stratified by C1qA, C1qB or C1qC expression. Only a few gene sets with high NOM and FDR values are shown.

In view of the fact that the levels of C1qA, C1qB, and C1qC expression are positively linked to OS patient survival, the high and low C1qA, C1qB, and C1qC expression groups were compared with the median group by GSEA. Both the Hallmark and C7 datasets showed that the groups with high C1qA, C1qB or C1qC expression levels were remarkably enriched in immune gene sets, indicating their immune functions, such as allograft rejection, the complement response, and the basic response, were more active than those in the low expression groups (**Figures 6A–F**). It is suggested that C1qA, C1qB and C1qC may be potential indexes that reflect the status of the TME.

### Correlation Analysis of the Levels of C1qA, C1qB or C1qC Expression and TICs

**Figure 7:**
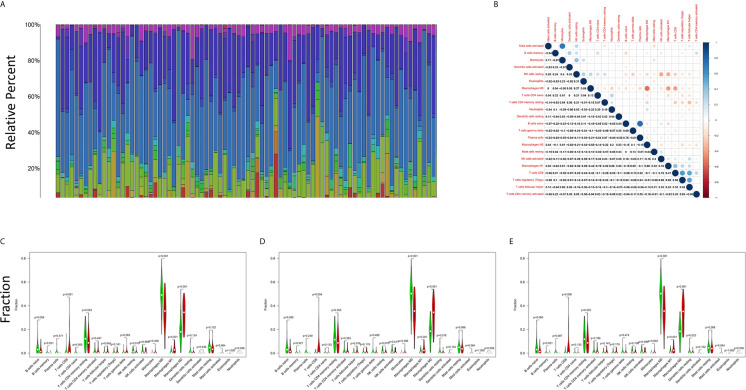
TIC profiles of tumor samples from OS patients and correlation analyses. **(A)** The proportions of 22 types of TICs among the tumor samples from OS patients are shown in the bar chart. Sample IDs are used as the names of column. **(B)** The correlations among the 22 types of TICs in the different OS samples are indicated in the heatmap with the p values (number in each box) of the correlations between two cells shown. The corresponding correlation values (shade of each colored box) are displayed, and significance was evaluated using the Pearson correlation coefficient. **(C–E)** The differences in 22 kinds of TICs between the high and low C1qA, C1qB and C1qC expression groups are shown in the violin plot. The Wilcoxon rank-sum was applied to test significance. The TIC names are shown on the X-axis, and the contents are indicated on the Y-axis.

**Figure 8:**
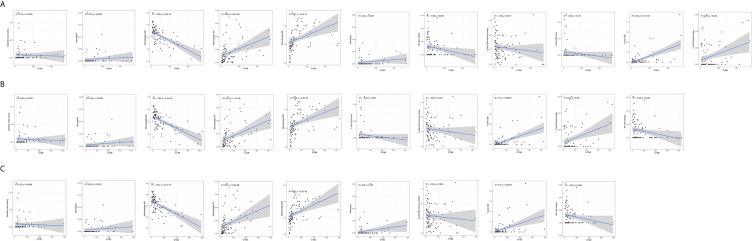
Correlations between C1qA, C1qB, or C1qC expression levels and TIC proportions. **(A–C)** The scatter plots show the correlations between C1qA, C1qB or C1qC expression levels and TIC proportions (*p <* 0.05). The proportion tropism of the immune cells and C1qA, C1qB, and C1qC expression levels are indicated by a fitted linear model in each plot, as shown by the blue line. Significance was tested using the Pearson correlation coefficient.

**Figure 9:**
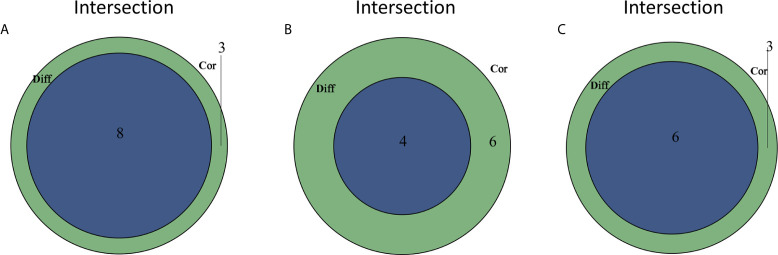
TICs related to the expression of C1qA, C1qB or C1qC. **(A)** Venn diagram showing the 8 TICs related to C1qA expression. **(B)** Four TICs related to C1qB expression. **(C)** Six TICs related to C1qC expression. These TICs were determined jointly from the differences and correlation tests shown by the violin and scatter plots.

The CIBERSORT algorithm was applied to analyze the proportions of tumor-infiltrating immune subsets to further confirm the correlations between the TME and C1qA, C1qB, and C1qC expression levels. Construction of 22 kinds of immune cell profiles in OS samples was performed (**Figure 7A**), and the correlations with TICs were calculated (**Figure 7B**). The differences and correlations between the C1qA, C1qB, and C1qC expression levels and the proportions of TICs were analyzed (**Figures 8** and **9**). The results showed that there were 8 kinds of C1qA expression-related TICs (**Figure 9A**). Among them, three kinds of TICs were positively associated with C1qA expression, including M1 macrophages, M2 macrophages, and CD8+ T cells. Five types of TICs, including M0 macrophages, resting NK cells, naive CD4+ T cells, and resting memory CD4 T cells, were negatively correlated with C1qA expression. Four types of TICs were linked to C1qB expression (**Figure 9B**); three kinds of TICs were positively associated with C1qB expression, including M1 macrophages, M2 macrophages, and CD8+ cells. A negative correlation between M0 macrophages and C1qB expression was found. Six types of TICs were linked to C1qC expression (**Figure 9C**). Among the types, 4 were positively associated with C1qC expression, including M1 macrophages, M2 macrophages, resting T cells, and CD8+ T cells. Two types of TICs were negatively correlated with C1qC expression, including M0 macrophages and resting memory CD4 T cells. However, the levels of M1 macrophages, M2 macrophages, and CD8+ T cells were positively associated with the expression levels of C1qA, C1qB and C1qC. These results further indicate the influence of C1qA, C1qB and C1qC expression levels on TME immunoactivities.

## Discussion

The TME has important effects on tumorigenesis and tumor development. Studies have shown that TME remodeling may promote the transformation of lung adenocarcinoma from a developmental status to an inhibitory status (17). In the TME literature related to OS, we found that scholars have explored the relationship between the immune score of TICs and the survival rate in OS patients, and PPARG, IGHG3 and PDK1 were used as prediction targets (12). The immune score has certain reference value for clinicians in evaluating the prognosis of OS patients and selecting appropriate immunotherapy targets. However, the associations between the targeted gene expression and clinicopathological characteristics of OS patients and TICs in the tumor microenvironment have not been reported. In our present study, a series of bioinformatic analyses using the TCGA and TARGET databases were performed to identify the genes related to the TME that can improve the prognosis of OS patients. Ultimately, C1qA, C1qB and C1qC were identified as the main indexes for predicting the prognosis of OS and remodeling of the TME. We will focus on the relationship between target gene expression and the TME to provide a new therapeutic target for OS immunotherapy.

According to the results of the study, we concluded that TME immune and matrix components contribute to OS patient prognosis. In particular, the proportions of TME immune components are remarkably linked to invasion and metastasis in OS patients. The importance of uncovering the interaction between immune and tumor cells in OS is emphasized by these results. Studies have also confirmed that TICs are tightly associated with the progression and prognosis of OS (18). These associations provide a new theoretical basis for the development of more effective immunotherapy programs. Immunotherapy is a potentially valuable therapeutic strategy to treat human malignancies. In recent years, OS immunotherapy has made great progress (19). First, obvious clinical effects have been achieved with cytokines, including interleukin-2 (IL-2) and interferon (IFN), used for sarcoma immunotherapy. However, high-dose IL-2 treatment is likely to cause a series of adverse reactions, so its use is limited to some extent (20). Subsequently, immunotherapies such as vaccines, adoptive cell transfers and immunoassay blockers have been applied in clinical research, and certain research results have been achieved (21, 22). However, in recent years, there have been no major breakthroughs that improve the clinical therapeutic effect on OS. Therefore, actively exploring potential therapeutic targets involved in TME remodeling is of great significance to provide candidate regimens for OS immunotherapy.

First, we studied the correlations between TME immune and matrix components and the survival rate of OS patients. The results showed that the levels of immune cells and stromal cells were significantly linked to the overall survival rate of OS patients. High-score and low-score samples were compared and analyzed, and 155 DEGs were identified for determination of exact gene profile changes in TME immune and matrix components. Most of these genes were upregulated in the TME, and these DEGs might be important factors in the change in the TME state. Subsequently, these DEGs were found to generally map to immune-related terms or disease maps through GO and KEGG enrichment analyses. These studies indicate that immune factors are a major feature of the OS tumor microenvironment. To further uncover the underlying endogenous mechanism, the construction of a PPI network and univariate Cox regression analysis based on OS patient survival were carried out, and key genes that affect patient survival were identified. The relationships between the differential expression of key genes and OS patient survival were also analyzed, and the results indicated that C1qA, C1qB, and C1qC differential expression were positively linked to the overall survival of OS patients. The five-year survival rate of the high expression subgroup in each of these three groups was above 75%, which was markedly higher than that of the low expression subgroup. Bandini (23) also confirmed that C1qA, C1qB and C1qC expression levels were positively linked to a good prognosis in breast cancer patients using *in vivo* investigation of C1q-deficient mice.

The complement system is an important arsenal of the innate immune system against pathogens as well as cancer. The complement system is initiated in response to the tumor-associated antigens and leads to increased deposition of complement activation fragments on the surface of the tumor (24).The first recognition subcomponent C1q, belonging to the C1q/Tumor Necrosis Factor superfamily, is important in the classical pathway of the complement system. In a complement-dependent or complement-independent manner, C1q can perform various immune and nonimmune functions. Additionally, recognition of various ligands and pathogens for clearance *via* effector mechanisms such as opsonization and the inflammatory response can be performed by C1q through modulation of innate immunity (25). C1q is highly expressed in various human tumor microenvironments (26). For example, in prostate cancer cells, C1q induces apoptosis by activating the tumor inhibitor WOX1 (27). C1q has been shown to have protective effects that limit the progression of cancer in BALB-neuT mice with breast cancer (28). However, in malignant pleural mesothelioma, C1q promotes tumor cell proliferation and migration and aggravates tumor development (29). In addition, C1q can activate the classical complement pathway to aggravate disease progression in the later stage of inflammatory disease, but it plays a protective role in the early stage of inflammatory disease development (30). Therefore, C1q may play dual survival-promoting and apoptosis-inducing regulatory roles in tumors. The potential mechanism of this dual characteristic of C1q may be determined by various factors, such as the type of tumor cells, the expression of immune genes, the nature of infiltrating immune cells, the degree of infiltration and the ability of infiltrating immune cells to synthesize C1q (and/or other complement components) locally. To clarify the roles of C1q in the occurrence and development of OS, the relationships between C1qA, C1qB, or C1qC expression levels and clinicopathological features (including age, histologic response, percent necrosis at definitive surgery, primary site progression, and primary tumor site) in OS patients was analyzed. The results demonstrated that the C1qA, C1qB and C1qC gene expression levels were higher in the OS patients with a percent necrosis at definitive surgery less than 50%. No significant correlations were found between the other clinical features and the expression of C1qA, C1qB or C1qC. Therefore, C1qA, C1qB and C1qC differential expression is closely related to the degree of tumor necrosis, plays a role in inhibiting tumor development to a certain extent. Some studies have found that human bone osteosarcoma epithelial cells (U2-OS) can activate the complement system by pooled normal human serum (31). This study presents a direct linkage of the complement system and angiogenesis in an *in vitro* cancer cell model, which could be useful in elucidating the relationship between the complement system and tumors necrosis and the underlying mechanisms.

To date, three “canonical” pathways of complement activation including the classical, alternative and lectin pathways have been identified. There is growing evidence that complement pathways can be activated in a variety of ways, depending on the triggering factors and the unique microenvironment or pathophysiological environment (32). Of course, complement may also play a tumor-promoting function that is unrelated to the activation of the canonical pathways. Based on previous GO and KEGG enrichment analysis results, we already knew that the overall function of DEGs seemed to map to immune-related activities, indicating that immune factors are a major feature of the TME. However, the immunomodulatory mechanism and actions of C1qA, C1qB and C1qC in osteosarcoma are not clear.

C1q receptors are expressed in a wide range of cells, including those that do not participate in the immune response. Nevertheless, the interaction of C1q with its receptor triggers various cellular responses, and these responses are suspected to involve certain signaling pathways (33). Therefore, we tried to further study the use of C1qA, C1qB, and C1qC through GSEA and evaluation of the correlations between immune regulation and differential expression. The results showed that the high C1qA, C1qB, and C1qC expression groups were all remarkably enriched in signaling pathways related to active immune functions, such as allograft rejection, the complement response, and the inflammatory response. However, with decreased differential expression of the C1qA, C1qB, and C1qC genes, the signaling pathway enrichment was significantly reduced. Obviously, the roles of the complement molecules C1qA, C1qB, and C1qC in inhibiting OS development are still related to antibody-mediated immunotherapy, which provides certain help for defining new treatment options.

Macrophages are reported to be the key effectors in the process of complement activation. Macrophages, the initial line of defense in the immune system, are specifically designed to initiate proper immune responses by responding to infectious microorganisms and normal or altered self-antigens. The CIBERSORT algorithm was applied to analyze the proportions of tumor-infiltrating immune types, and the construction of 22 immune cell profiles for OS samples was carried out. The results showed that the high expression groups for the three C1q chains (C1qA, C1qB, and C1qC) differed significantly from the corresponding low expression groups. In addition, M1 macrophages, M2 macrophages and T cells were positively correlated with the differential expression of the three C1q chains (C1qA, C1qB, and C1qC). M0 macrophages were negatively associated with the expression of the three C1q chains (C1qA, C1qB, and C1qC). These results suggest that the differential expression of the three C1q chains (C1qA, C1qB, and C1qC) is related to the levels of polarized M1 macrophages, polarized M2 macrophages and CD8+ T cells in the TME and is also a key indicator for improving the prognosis of OS. The polarization of macrophages is directed by the complement component C1q, and proresolution macrophages are generated, which can promote the clearance of apoptotic cells, diminish the production of proinflammatory cytokines, and increase anti-inflammatory cytokine production (34). Additionally, in response to tissue injury, macrophage-synthesized C1q may be an important effector driving the resolution of inflammation independent of other complement components. Numerous potentially beneficial interactions between C1q and phagocytes were identified *in vitro* in previous studies, including promotion of cellular and molecular debris phagocytosis and anti-inflammatory macrophage polarization. The prevention of autoimmunity may also be supported by these interactions (35). In addition, some studies have confirmed that C1q can regulate the mitochondrial metabolism of CD8+ T cells to inhibit responses to autoantigens, and there is a close relationship between C1q and CD8+ T cells (36). Moreover, CD8+ T cells can initiate autoimmunity and play a positive role in the prognosis of OS. The activation status (M1/M2) of macrophages may cause dual effects on tissue damage in neuroinflammatory diseases. M1 macrophages are reported to damage neurons, while M2 macrophages can regenerate and repair neurons (37). This conclusion contradicts our research. Therefore, the mechanism underlying the role of phagocytes in OS needs to be further confirmed by basic research.

In summary, we found for the first time through bioinformatics that the differential expression of the three C1q chains (C1qA, C1qB, and C1qC) is closely related to the survival prognosis, pathological features and TME of OS, which can provide some help for improving OS immunotherapy and clinical prognosis. Since the relationship between the complement system and tumors remains unclear, a complete theoretical framework has not emerged. Further research is needed to uncover the regulatory mechanism and develop new immunotherapeutic strategies.

## Data Availability Statement

The datasets presented in this study can be found in online repositories. The names of the repository/repositories and accession number(s) can be found below: https://portal.gdc.cancer.gov, The Cancer Genome Atlas and https://ocg.cancer.gov/programs/target, TARGET database.

## Ethics Statement

Written informed consent was obtained from the individual(s), and minor(s)’ legal guardian/next of kin, for the publication of any potentially identifiable images or data included in this article.

## Author Contributions

L-hC, J-FL, and YL came up with the design and conception. The data analysis and visualization were conducted by L-hC, X-yH, and C-Z. The original writing of the draft and its editing were by L-hC and H-hZ. All authors contributed to the article and approved the submitted version.

## Conflict of Interest

The authors declare that the research was conducted in the absence of any commercial or financial relationships that could be construed as a potential conflict of interest.

## Supplementary Material

Supplementary Figure 1 Relationships between CD163 expression and survival time in patients with OS.

Supplementary Table 1 TICs co-determined by difference test and correlation test.
