# Supplementary figures and images for: Complement C1q (C1qA, C1qB, and C1qC) May Be a Potential Prognostic Factor and an Index of Tumor Microenvironment Remodeling in Osteosarcoma
Source: Front Oncol. 2021 May 17;11:642144. doi: 10.3389/fonc.2021.642144 (PMC8166322; doi:10.3389/fonc.2021.642144)

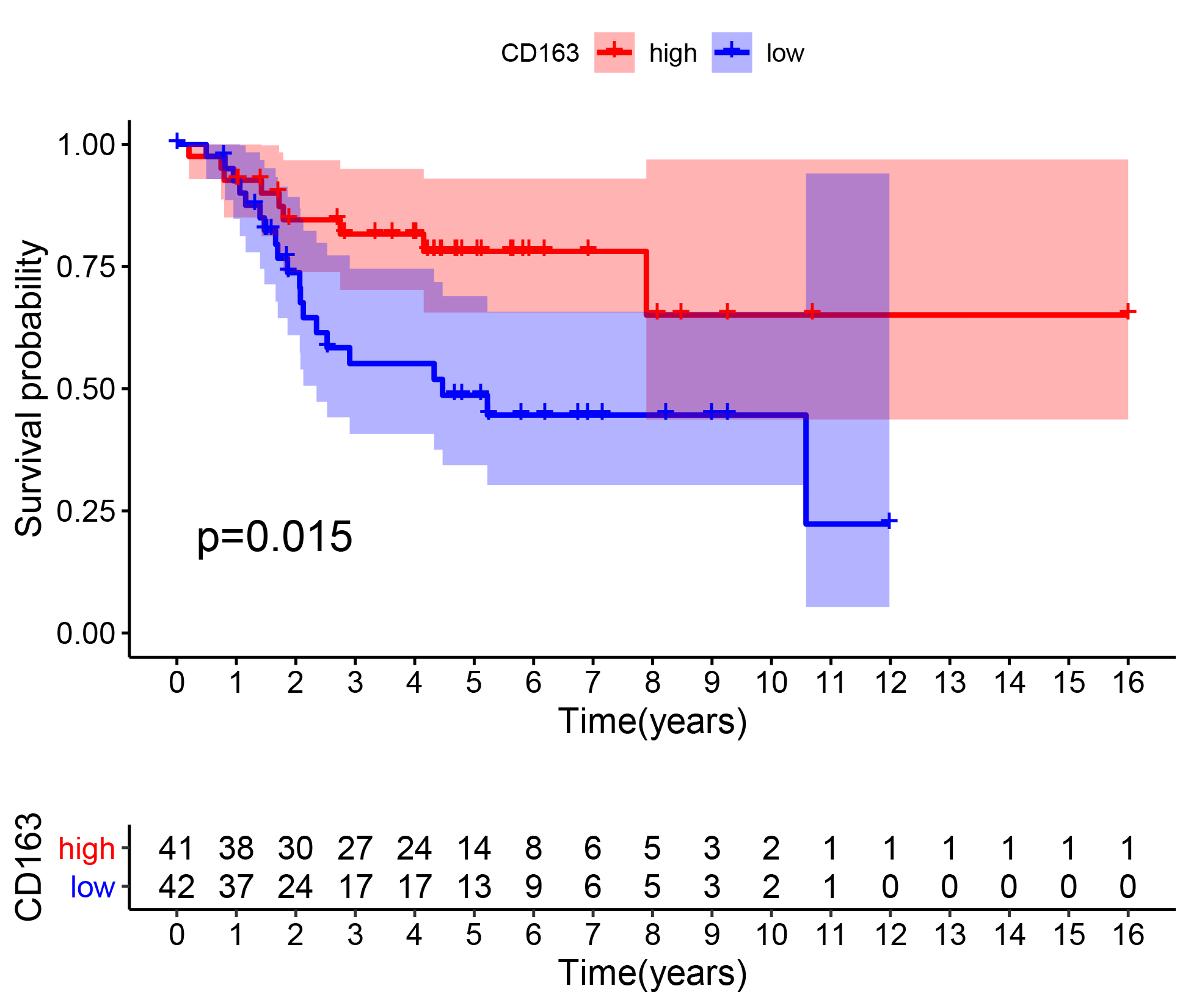

Supplement: Supplementary Figure 1 — Relationships between CD163 expression and survival time in patients with OS. [file Image_1.tif]
