# Supplementary material for: Complement C1q (C1qA, C1qB, and C1qC) May Be a Potential Prognostic Factor and an Index of Tumor Microenvironment Remodeling in Osteosarcoma
Source: Front Oncol. 2021 May 17;11:642144. doi: 10.3389/fonc.2021.642144 (PMC8166322; doi:10.3389/fonc.2021.642144)
Supplement: Supplementary Table 1 — TICs co-determined by difference test and correlation test. [file Table_1.pdf]

Supplement Table 1.TICs co-determined by difference test and correlation test

Gene	TICs	Correlation Test (p-value)	Difference test (p-value)
C1qA	T cells CD8	0.42 （< 0.001）	0.001
	T cells CD4 naive	-0.26 （0.015）	0.005
	T cells CD4 memory resting	-0.26 （0.017）	0.023
	T cells follicular helper	0.23 （0.038）	0.045
	NK cells resting	-0.29 （0.007）	0.009
	Macrophages M0	-0.63 （< 0.001）	< 0.001
	Macrophages M1	0.58 （< 0.001）	< 0.001
	Macrophages M2	0.67 （< 0.001）	< 0.001
C1qB	T cells CD8	0.4 （< 0.001）	0.003
	Macrophages M0	-0.62 （< 0.001）	< 0.001
	Macrophages M1	0.57 （< 0.001）	< 0.001
	Macrophages M2	0.63 （< 0.001）	< 0.001
C1qC	T cells CD8	0.4 （< 0.001）	0.005
	T cells CD4 memory resting	-0.24(0.025)	0.019
	Macrophages M0	-0.61 （< 0.001）	< 0.001
	Macrophages M1	0.57 （< 0.001）	< 0.001
	Macrophages M2	0.65 （< 0.001）	< 0.001
	Dendritic cells resting	0.32 （< 0.001）	0.023
